# Esophageal microbiome in active eosinophilic esophagitis and changes induced by different therapies

**DOI:** 10.1038/s41598-021-86464-z

**Published:** 2021-03-29

**Authors:** E. J. Laserna-Mendieta, J. A. FitzGerald, L. Arias-Gonzalez, J. M. Ollala, D. Bernardo, M. J. Claesson, A. J. Lucendo

**Affiliations:** 1 Department of Gastroenterology, Hospital General de Tomelloso, Vereda de Socuéllamos, s/n, 13700 Tomelloso, Ciudad Real, Spain; 2 Instituto de Investigación Sanitaria de La Princesa, Madrid, Spain; 3 Clinical Laboratory, Hospital Universitario de La Princesa, Madrid, Spain; 4 School of Microbiology, University College Cork, Cork, Ireland; 5 APC Microbiome Ireland, Cork, Ireland; 6 Department of Pathology, Hospital General La Mancha Centro, Alcázar de San Juan, Spain; 7 Mucosal Immunology Lab, Instituto de Biología Y Genética Molecular (IBGM), Universidad de Valladolid-CSIC, Valladolid, Spain; 8 Centro de Investigación Biomédica en Red Enfermedades Hepáticas Y Digestivas (CIBERehd), Madrid, Spain

**Keywords:** Oesophageal diseases, Gastroenterology

## Abstract

Eosinophilic esophagitis (EoE) is a chronic, immune-mediated inflammatory esophageal disease triggered by food antigens. Cumulative evidence supports the implication of microbiota and the innate immune system in the pathogenesis of EoE. Changes in the esophageal microbiome were investigated by applying 16S rRNA gene sequencing on esophageal biopsies of adult patients with active EoE at baseline (n = 30), and after achieving remission with either proton pump inhibitors (PPI, n = 10), swallowed topical corticosteroids (STC, n = 10) or food-elimination diets (FED, n = 10). Ten non-EoE biopsies were also characterized as controls. Compared to controls, no differences in alpha (intra-sample) diversity were found in EoE microbiota overall. However, it decreased significantly among patients who underwent FED. As for beta (inter-sample) diversity, non-EoE controls separated from EoE baseline samples. Post-treatment samples from patients treated with PPI and FED had a more similar microbiota composition, while those receiving STC were closer to controls. Differential testing of microbial relative abundance displayed significant changes for *Filifactor, Parvimonas* and *Porphyromonas* genera. Analysis of predicted functions indicated alterations in metabolic pathways and abundance of sulphur-cytochrome oxidoreductases. Our findings demonstrate changes in microbiota associated with EoE, as well as a treatment effect on the microbiome.

## Introduction

Eosinophilic esophagitis (EoE) is a chronic, immune-mediated inflammatory disease of the esophagus, which consists of a non-IgE-mediated response to antigens present in the diet. EoE typically manifests clinically as symptoms of esophageal dysfunction, and histologically through an eosinophil-predominant inflammation restricted to the esophagus^1^. The incidence and prevalence of EoE has sharply increased in the last few years, being currently 7.7 person-years and 42.2 cases per 100,000 inhabitants for adults^2^, without a clear causative explanation. Evidence shows that environmental factors could be the predominant cause to explain such a boost in disease rate^3^. Among them, some hypotheses have pointed to esophageal dysbiosis as a trigger of EoE pathology^3^, and early-life environment factors affecting the microbiota might contribute to EoE susceptibility^4^. However, the characterization of esophageal microbiota through culture-independent techniques is a relatively unexplored field, with a first preliminary study as recent as 2004 identifying the genus *Streptococcus* as the most abundant in the esophagus, followed by *Veillonella* and *Prevotella*^5^. A few years later, the first study addressed to analyze alterations in the esophageal microbiota associated with esophageal diseases, specifically in patients with Barrett’s esophagus and reflux esophagitis, showed a dysbiosis characterized by a higher content of gram-negative anaerobes or microaerophiles^6^. More recently, several examples of alterations in the esophageal microbiome have been reported in association with major esophageal diseases, including cancer and EoE^7,8^. Nevertheless, studies about esophageal microbiota in EoE are scarce and still require further research to confirm and extend their findings^9–11^. Recently, new studies in saliva^12^ and stool^13^ have revealed further changes in the microbiomes of EoE patients, supporting the idea that additional studies to elucidate microbiota disturbances in EoE are necessary.

Here, we aimed to investigate differences in the esophageal microbiota between pre-treatment and post-treatment biopsies, where subjects successfully achieved remission through treatment either with proton pump inhibitors (PPI), swallowed topical costicosteroids (STC), or use of empirical food elimination diets (FED). We compared microbiota diversity and composition among these groups and also to non-EoE controls. Hence, and to our knowledge, this is the first microbiota research in EoE differentiating among the three major treatment options currently used to induce EoE remission.

## Results

### Sample processing before data analysis

Esophageal biopsies were obtained via upper endoscopy of 30 adult patients diagnosed with EoE, at two different time points (at baseline and after achieving disease remission with successful treatment with PPI, STC or FED), and from 10 non-EoE controls. The demographical and clinical characteristics of the individuals included in the study are described in Table 1.

**Table 1:** Demographic and clinical characteristics of the subjects included in the study.

		n	Gender (% male)	Age: Mean ± SD (range)	Peak of eos (Pre-Tx): Median (IQR)	Peak of eos (Post-Tx): Median (IQR)	Type of Tx (EoE)/Main cause for endoscopy (controls)
EoE patients		30	90%	27.7 ± 10.9 (16–45)	72 (49–98)	0 (0–3)	
EoE patients by treatment	FED	10	90%	25.1 ± 11.4 (16–38)	100 (80–158)	3 (1–8)	2FED: 50%; 4FED: 40%; 6FED: 10%
	PPI	10	80%	30.3 ± 10.2 (17–45)	56 (43–60)	3 (0–6)	Omeprazole: 90%; Lansoprazole: 10%
	STC	10	100%	27.6 ± 11.6 (16–44)	66 (40–108)	0 (0–0)	Budesonide: 90%; Fluticasone: 10%
Non-EoE controls		10	50%	35.8 ± 12.7 (16–53)	ND	–	Dyspepsia: 60%; Dysphagia: 20%; Iron deficiency: 40%

EoE: eosinophilic oesophagitis; eos: eosinophils; n: number of subjects; Tx: treatment; IQR: interquartile range; FED: food-elimination diet; PPI: proton pump inhibitors; STC: swallowed topical corticosteroids; ND: not determined (2 controls had no detectable eosinophils, 8 controls had no histological alterations in the oesophageal epithelium).

Therefore, 70 samples were initially processed for 16S V4 rRNA amplicon library construction (using 515F and 806R primers) and sequencing, jointly with 3 negative controls. One post-treatment sample failed during 16S rRNA amplicon library generation and it was not sequenced. After quality checking, trimming processes, and an additional decontamination step (see methods) samples with less than 500 reads were removed, excluding four additional samples: two pre-treatment and two post-treatment. Consequently, a final number of 65 samples were used for microbiota analysis (mean reads per sample: 9575 + /−6688 reads; Supplementary Table 1).

### Intra-individual diversity

**Figure 1:**
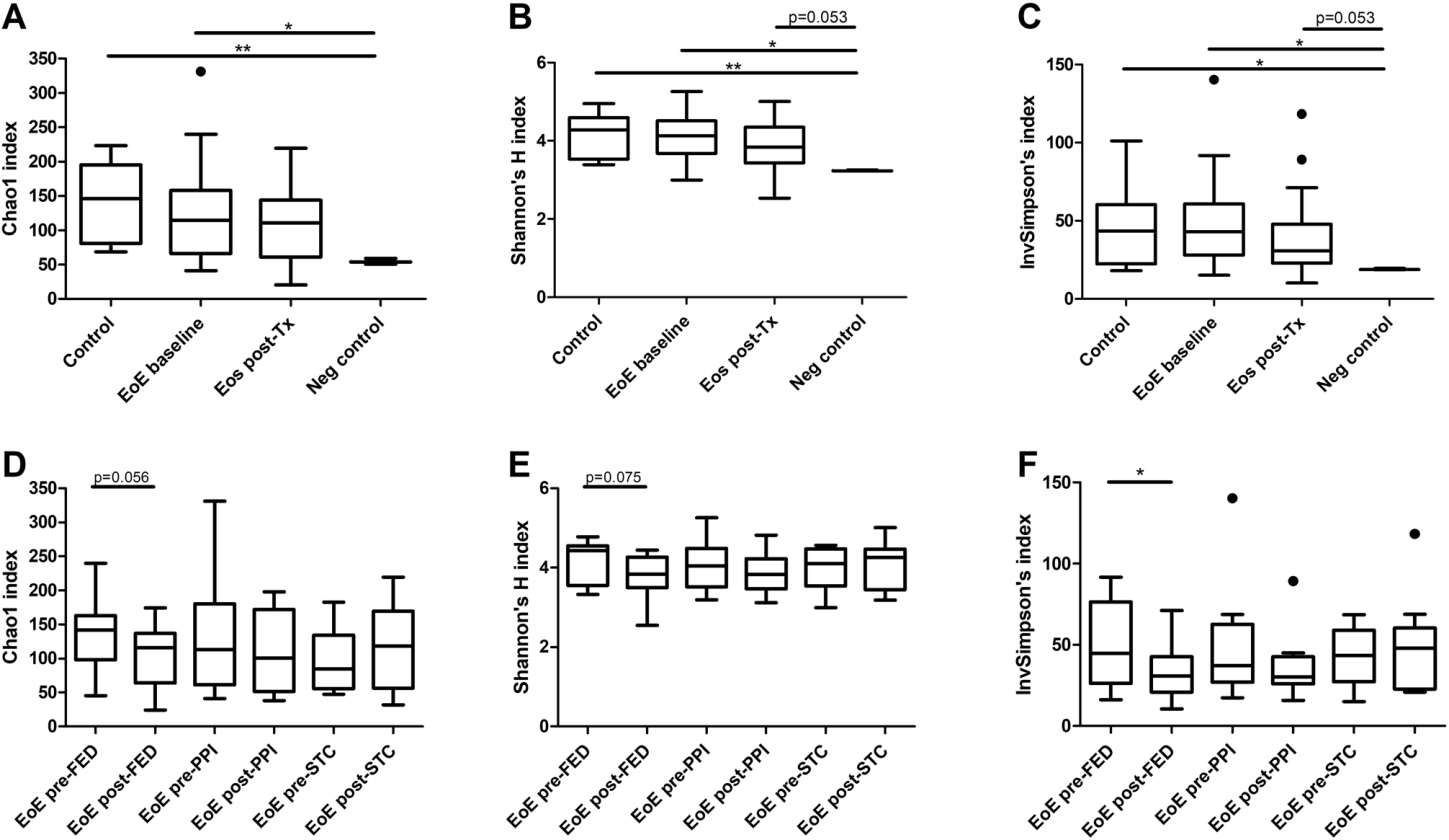
Comparison of oesophagus microbiota alpha-diversity across groups. Tukey plots showing alpha-diversity for Chao1 (**A** and **D**), Shannon’s H (**B** and **E**) and inverse Simpson’s (**C** and **F**) indices. Comparison of control subjects, baseline and post-treatment EoE patients, and negative controls for each of the indices analyzed (**A**–**C**). Comparison of paired pre- and post-treatment samples divided by treatment for each of the indices analyzed (**D**-**F**). *p < 0.05, **p < 0.01. EoE: eosinophilic oesophagitis; EoE post-Tx: EoE patients after treatment; Neg control: negative controls for 16S amplification; FED: food elimination diet; PPI: proton pump inhibitors; STC: swallowed topical corticosteroids.

Comparison of rarefied microbiota alpha (intra-sample) diversity across groups was evaluated using three indices: the Chao1 index, the Shannon’s H index, and the inverse Simpson’s index. No significant differences were detected in Student’s t-tests for any of the indices between samples from control subjects and baseline EoE patients, nor between control and post-treatment EoE samples overall. As expected, alpha-diversity for the three studied groups was higher than for negative controls, although it did not reach statistical significance for post-treatment samples (Fig. 1A–C). Paired Student’s t-test or Wilcoxon test displayed no significant differences for alpha-diversity between baseline and post-treatment samples for any of the indices, although diversity was slightly higher in the baseline group in all indices (Supplementary Fig. 1A–C).

When baseline samples were classified according to the specific therapy the patients received, no differences in alpha-diversity were found among them using one-way ANOVA (Supplementary Fig. 1D–F). When post-treatment samples were also classified according to therapy, a significant reduction in inverse Simpson’s (*p* = 0.049) index was detected in paired t-test exclusively for samples from patients treated with a FED, while *p*-values were close to significance for Chao1 and Shannon’s H indices (0.056 and 0.075, respectively) (Fig. 1D–F).

In addition, we calculated Faith’s phylogenetic diversity which also showed significant differences between the three groups of samples and negative controls. However, no differences were detected between groups when samples were divided according to the treatment received (Supplementary Fig. 2).

Taken together, inflammation in EoE did not significantly change the alpha-diversity of esophageal microbiota compared to non-EoE subjects, and only patients who underwent FED experienced a decrease in microbial diversity, likely due to the food restrictions itself.

### Inter-individual diversity and clustering

**Figure 2:**
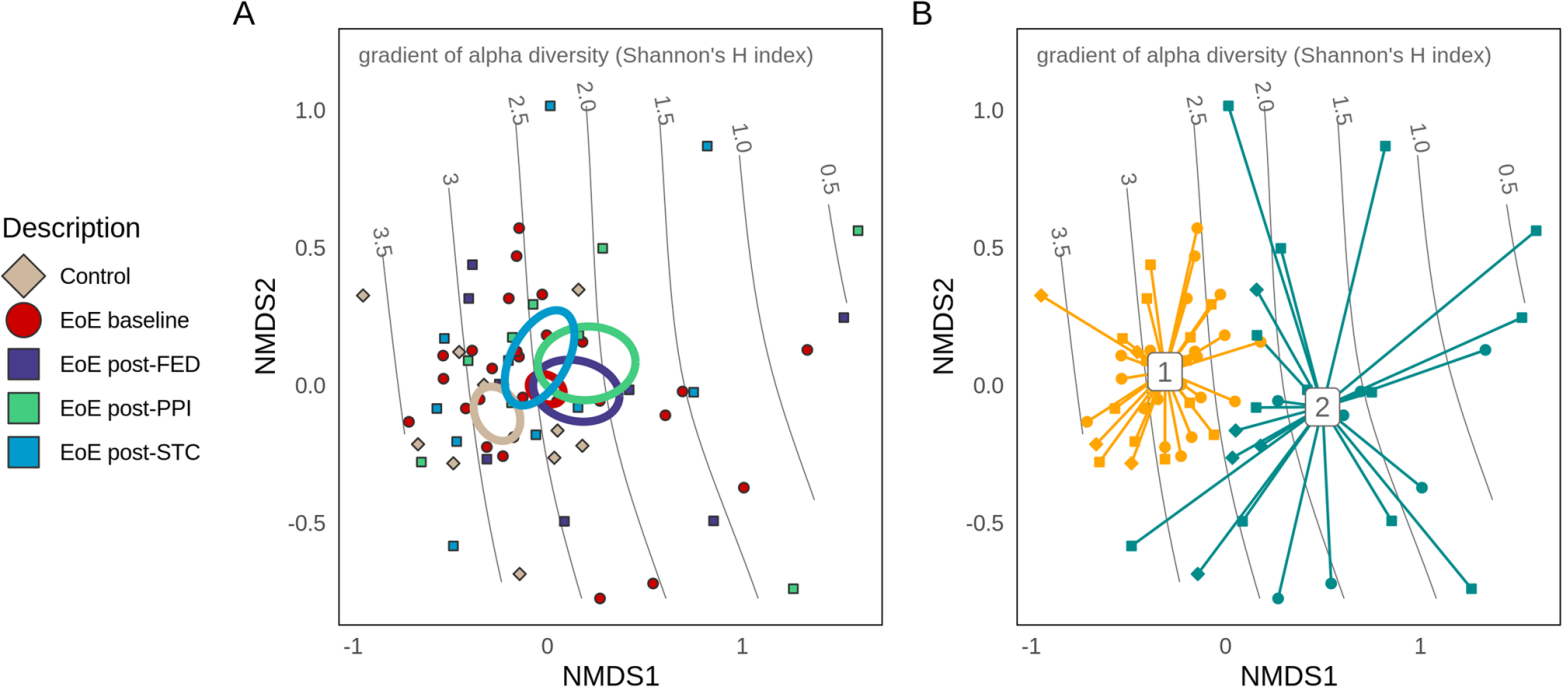
Comparison of microbiome beta-diversity across conditions based on ASV features using non-metric dimensional scaling (NMDS). Ellipses representing centroid position for each group, showing the distribution of samples and groups along the primary axis (NMDS1), closely aligned with a gradient in microbial alpha-diversity (**A**); Dirichlet multinomial mixture (DMM) groups defined through differences in patterns of abundance were well separated along the primary X axis and gradient in alpha-diversity (**B**).

Comparison of microbiome beta (inter-sample) diversity was performed on Amplicon Sequence Variant (ASV) features using non-metric dimensional scaling (NMDS) of the Bray–Curtis dissimilarity, a well-established multivariate method for relating experimental conditions to underlying patterns in microbial community structure. NMDS analysis is shown in Fig. 2A, which additionally maps the gradient of alpha-diversity across samples within the study, where sample location reflects the corresponding alpha-diversity value. Despite overall similarities in composition, NMDS placed treatment groups along a primary (x) axis, while sample position along the gradient independently corresponds with the decline in alpha-diversity (axis NMDS1, Fig. 2A). It was observed that the centroid (i.e. the average location) of control samples was located at a more microbially diverse point than other groups along the gradient of alpha-diversity, closest to the centroid for post-STC and outside of standard-error confidence limits from centroids for EoE baseline, post-PPI, and post-FED. The majority of EoE baseline samples were also positioned in areas of higher alpha-diversity, while post-PPI and post-FED groups were dispersed across a range of lower alpha-diversities.

Further independent classification of samples based on their frequencies of population abundance (Dirichlet-multinomial mixture models, DMM) resolved the samples into two groups which showed significant differences in alpha diversity (*p* < 0.001, Supplementary Fig. 3B) and corresponded well with their locations both along the alpha diversity gradient, and along the primary NMDS1 axis (Fig. 2B). DMM group 1 contained a larger portion of baseline samples, but composition was otherwise balanced across treatment and control groups (Supplementary Fig. 3A).

Alternatively, the more classical analysis of beta-diversity by PCoA (based on Bray–Curtis index) was also performed using genus-agglomerated features. In agreement with the previous NMDS analysis, microbiota composition after PPI and FED therapies showed a high overlap between them, while a shift along the axis 1 was observed for EoE patients with STC therapy (Supplementary Fig. 4). In addition, baseline EoE samples displayed a shift along axis 3 compared to non-EoE controls.

**Figure 3:**
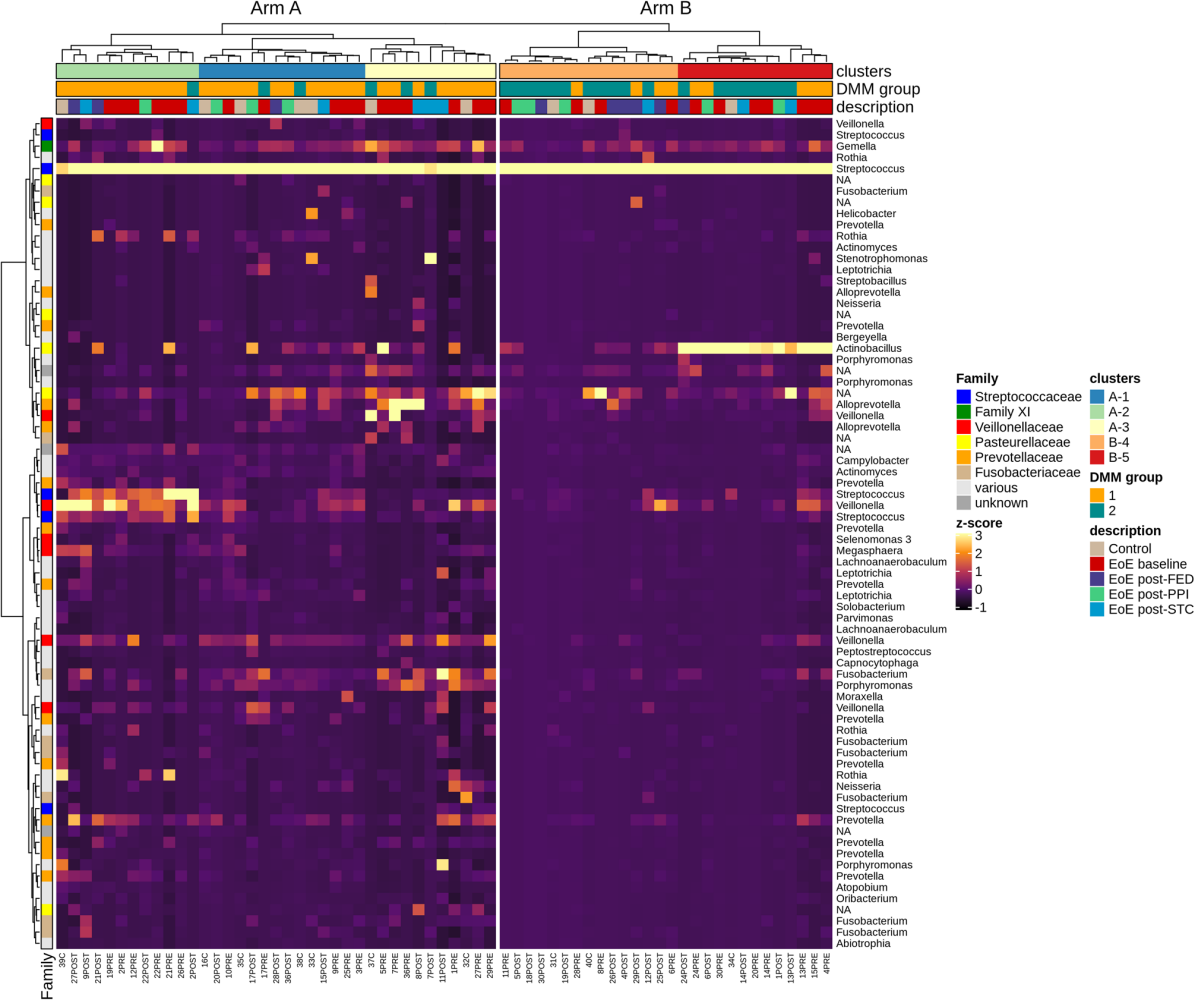
Heatmap and sample clustering. Z-score abundance heatmap with hierarchical Ward’s D2 linkage clustering based on Jaccard distances. The heatmap was created as a subset of features (74 out of 1004) with abundances above 800 reads. Colour codes were added to identify samples according to disease/treatment conditions, hierarchical cluster, and Dirichlet multinomial mixture (DMM) groups as defined in Fig. 2B.

Adaptive pruning of hierarchically-clustered Jaccard community dissimilarities using DynamicTree Cut package defined two community types (arms A and B; Fig. 3), containing five further subtypes (“clusters”) of community composition. Arm A (clusters 1, 2 and 3; 37 samples) comprised most of the control (7 of 10) and post-STC (6 of 9) samples, and was primarily (84%) composed of samples from DMM group 1 as identified above. In contrast, arm B contained clusters 4 and 5 (28 samples), predominantly (78%) falling within DMM group 2. When a 10,000-fold bootstrap of cluster assignments was implemented, post-STC samples still grouped separately from post-FED and post-PPI (Supplementary Table 2).

As such, analysis and clustering of microbial diversity identified two broad sample groups: one with a significantly higher alpha diversity, incorporating a greater proportion of EoE baseline samples; and another with overall lower alpha diversity and composed of an even mix of treatment types. These analyses also showed that baseline EoE samples displayed an esophageal microbiota composition that differed to some extent from that present in non-EoE subjects, with FED and PPI therapies leading to more similar esophageal microbiota compositions, while STC-treated patients appeared more similar to that of controls.

### Microbial composition

**Figure 4:**
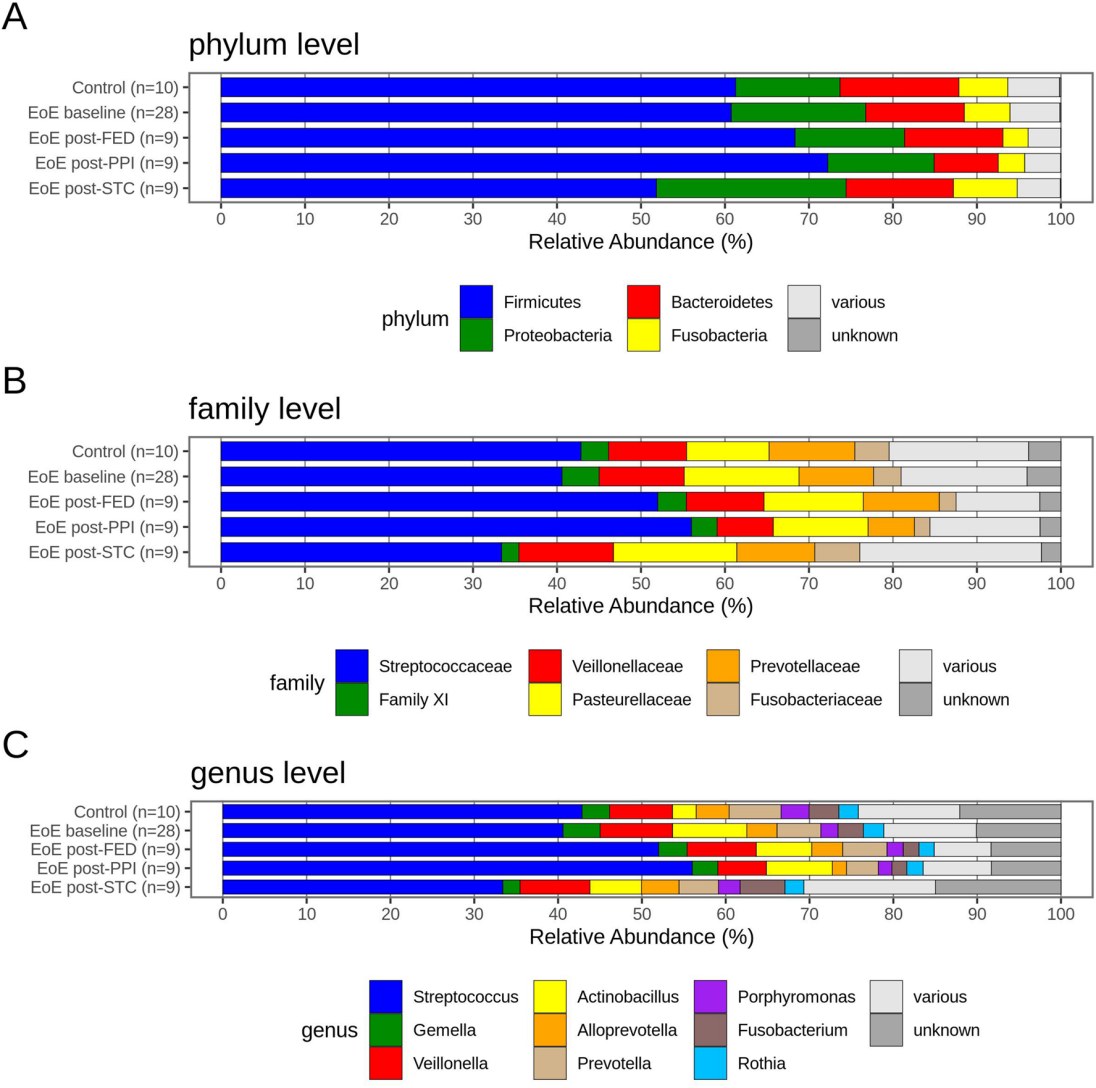
Relative bacterial abundance of the most represented phyla (**A**), families (**B**) and genera (**C**) in oesophageal biopsies classified within the following groups: control subjects, baseline EoE patients, and EoE patients according to treatment followed to achieve EoE remission.

Overall microbiota composition, annotated using the SILVA database for taxonomic identification, and grouped and summed to total abundance per condition for the main phyla, families and genera, is shown in Fig. 4. Firmicutes was the predominant phylum across all sequence reads (65%), followed by Proteobacteria (18%) and Bacteroidetes (9%) (Fig. 4A). Family *Streptococcaceae* was the most abundant in all conditions (50%) (Fig. 4B) and similarly, *Streptococcus* was also the most represented genus with a single ASV contributing sample abundances ranging from 33 to 56% (50% of all reads; Fig. 4C). No species accession was assigned to this dominant *Streptococcus* ASV, and a BLASTn search (NCBI 16S database) returned a number of exact, full-sequence (253 bp) matches with a number of *Streptococcus* species (*S. mitis, S. oralis, S. gordonii*, *S. periodonticum, S. gwangjuense, S. cristatus, S. infantis*).

Overall, no major differences were observed between groups. A slightly higher abundance of Proteobacteria and lower abundance of Bacteroidetes in controls was noticed compared with baseline samples from EoE patients. Patients treated with STC showed a lower abundance of Firmicutes and a relatively higher proportion of Proteobacteria, Bacteroidetes and Fusobacteria. Samples from patients treated with PPI therapy had on average a lower abundance of Bacteroidetes and Fusobacteria than EoE baseline, and held the highest compositional proportion of Firmicutes.

### Differential testing of microbial relative abundance

Differential analyses of microbial abundance were performed with abundances pooled at genus-level prior to a centre log-ratio (CLR) transformation, and using the Kruskal–Wallis test followed by the Wilcoxon signed-rank or Dunn’s test as appropriate for paired or independent data, respectively. Genera with significant changes for an adjusted *p*-value < 0.1 in post-hoc testing after Benjamini–Hochberg correction are described in Table 2. These changes were identified for *Filifactor, Parvimonas* and *Porphyromonas* genera, which were less abundant in baseline samples from EoE patients than from controls. *Filifactor* and *Porphyromonas* showed even a slightly lower abundance after treatment, while *Parvimonas* displayed a partial recovery after therapy that was not significant (adjusted *p*-value = 0.679, unadjusted *p* = 0.036).

**Table 2:** Differential testing of microbial relative abundance.

	Filifactor	Parvimonas	Porphyromonas
**Median (IQR)**			
Controls	0.12 (0.05–0.25)	0.38 (0.12–1.02)	3.0 (2.0–5.1)
EoE baseline	0 (0–0.13)	0.11 (0–0.22)	1.3 (0.6–3.0)
EoE post-treatment	0 (0–0.11)	0.22 (0–0.38)	0.9 (0.2–2.5)
Subjects with % > 0	31 (47.7%)	45 (69.2%)	60 (92.3%)
**BH-adjusted *** p***-values (FDR)**			
Controls *vs.* EoE baseline	0.090	0.003	0.047
Controls *vs.* post-Tx	0.025	0.042	0.006

Median and interquartile range (IQR) of each group for the three genera that showed significant differences and percentage of subjects (out of 65) in which each genus was detected. Output adjusted *p*-values (Benjamini–Hochberg correction) of the statistical comparisons are also provided. EoE: eosinophilic oesophagitis; post-Tx: EoE patients after treatment; BH: Benjamini–Hochberg correction; FDR: false discovery rate.

**Figure 5:**
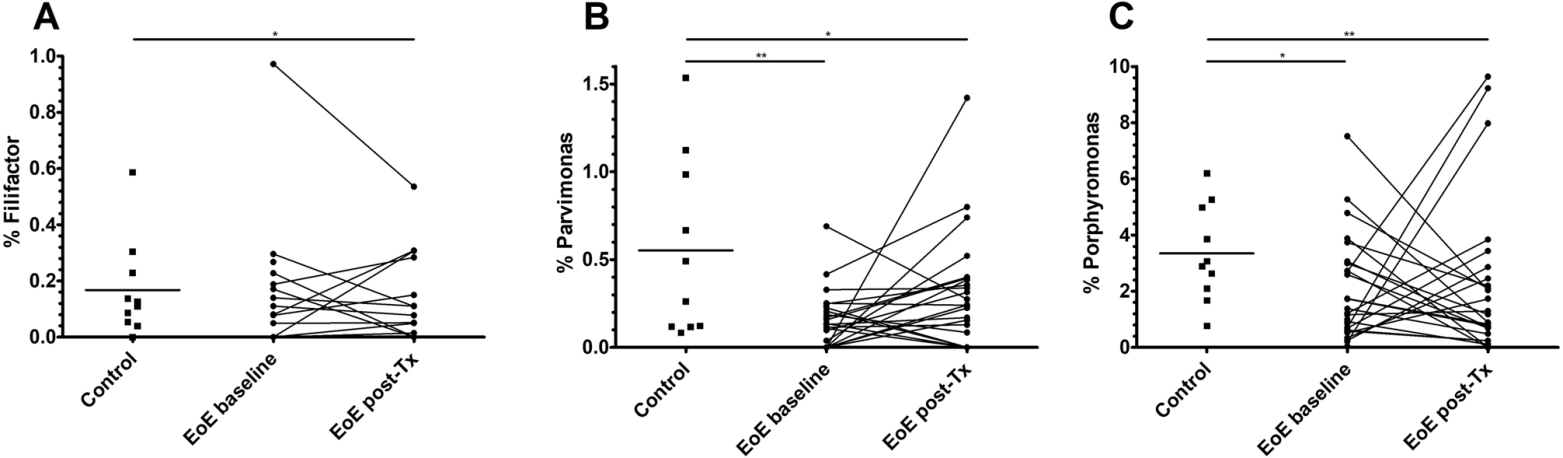
Dot plot representation for each condition of the relative abundance in oesophageal biopsies of bacterial genera *Filifactor* (**A**), *Parvimonas* (**B**) and *Porphyromonas* (**C**). EoE: eosinophilic oesophagitis; EoE post-Tx: EoE patients after treatment. *p < 0.05; **p < 0.01 (adjusted *p*-values).

Among these three genera, *Porphyromonas* was the most abundant one, ranging from 1 to 3% and being detected in 92% of the individuals. On the contrary, *Filifactor* and *Parvimonas* were less represented in the oesophageal microbiota (below 0.5%) and were detected in 48% and 70% of the subjects, respectively (Table 2 and Fig. 5).

### Differential abundance analysis for predicted metabolic content and pathways

The metabolic inference package PICRUSt2 enabled an analysis of predicted enzymatic functions and metabolic pathways of relevant changes beyond those of microbial taxonomy. Differential abundance analysis of enzymatic functions and metabolic pathways was performed following the same procedure as described for microbial relative abundance, but reporting changes with adjusted *p* < 0.1 for enzymes and with adjusted *p* < 0.25 for pathways (Table 3 and Supplementary Fig. 5).

**Table 3:** Differential abundance testing for predicted metabolic content and pathways.

	BH-adjusted * p*-values (FDR)				
	EC 1.8.2	PWY.5022	PWY.6470	GLUTORN.PWY	ARGSYN.PWY
Controls *vs.* EoE baseline	ns	0.248	ns	0.129	0.241
Controls *vs.* post-FED	ns	0.175	ns	ns	ns
Controls *vs.* post-PPI	ns	0.190	ns	ns	ns
Controls *vs.* post-STC	ns	ns	ns	0.229	ns
Pre-STC *vs.* post-STC	0.082	ns	ns	ns	ns
Post-STC *vs.* post-FED	0.048	ns	0.155	ns	ns
Post-STC *vs.* post-PPI	0.060	ns	ns	ns	ns

Only reactions (Enzyme Commision number; EC) and pathways (PWY) with at least one significant difference between groups according to adjusted *p* < 0.1 and *p* < 0.25 (Benjamini–Hochberg correction), respectively, are shown. BH: Benjamini–Hochberg correction; FDR: false discovery rate; EC 1.8.2: sulphur-cytochrome oxidoreductases; PWY.5022: 4-aminobutanoate degradation; PWY.6470: peptidoglycan biosynthesis V (β-lactam resistance); GLUTORN.PWY: L-ornithine biosynthesis I; ARGSYN.PWY: L-arginine biosynthesis II; EoE: eosinophilic oesophagitis; FED: food-elimination diet; PPI: proton pump inhibitors; STC: swallowed topical corticosteroids; ns: not significant (*p* > 0.1 for EC and *p* > 0.25 for PWY).

Predicted abundances of metabolic reactions (Enzyme Commission numbers; EC) were differentially tested among groups. Only oxidation/reduction of sulphur groups via a ferricytochrome acceptor (EC 1.8.2) was predicted to differ notably between treatments. PICRUSt2 assigned these functions to ASVs within the phyla Proteobacteria and Bacteroidetes, and in particular to ASVs in the genus *Pseudomonas* and a number of unidentified ASVs in the *Burkholderiaceae* family. An increase in EC 1.8.2 was observed in the post-STC group relative to EoE baseline (*p* = 0.082), post-PPI (*p* = 0.060), and post-FED groups (*p* = 0.048).

Secondly, differences in predicted metabolic pathway abundances were analyzed across samples, but were found to be relatively unaffected according to the adjusted *p*-values obtained (between 0.129 and 0.248). Small predicted differences in pathway abundance were seen for biosynthesis of the related amino acids arginine and ornithine (pathways ARGSYN and GLUTORN, respectively; higher EoE baseline than in controls), degradation of 4-aminobutanoate (pathway 5022; higher in controls than EoE baseline, post-PPI and post-FED), and peptidoglycan synthesis and β-lactam resistance (pathway 6470; higher in post-FED samples than after STC treatment).

Taken together, this suggests that metabolism of sulphur groups, 4-aminobutanoate, and ornithine/arginine, as well as cell wall synthesis and antimicrobial resistance could be affected by microbial changes present in patients with EoE, and that these alterations may be related to the treatment received.

## Discussion

A key role of microbiota in the development of several human diseases is widely accepted nowadays, associated with a proliferation of studies on the role of microbiota in the last decade^14^. However, the esophageal microbiota has not received as much attention as has been payed to other locations in the digestive system^15^, and thus its relation with esophageal diseases is still unclear. Our study was aimed to investigate microbiota changes in patients with EoE before and after treatment, applying modern next-generation sequencing (NGS) procedures and bioinformatic pipelines. To our knowledge, this is the first microbiota research in EoE differentiating the three major treatment options used for these patients.

Until now, only two studies have been published as full papers analyzing the microbiota present in the biopsies of patients with EoE^9,10^. As with our study, the total numbers of samples were around 70, so the consequent low number of samples in sub-groups was also a hurdle in the differential testing of microbiota between cohorts. Before comparing these prior works to outcomes in this study, it should be taken into account that we followed different procedures for 16S rRNA gene amplification/sequencing and bioinformatic analyses. As those studies were published in 2015, our study employed more recent technology for sequencing and more evolved bioinformatic tools for microbiota characterization, and differences in methodology are known to influence the observed microbial composition^16^.

Benitez and colleagues investigated pediatric EoE patients treated only with FED^9^, whereas the study cohort presented herein was composed of adult EoE patients treated with FED, PPI and STC. Benitez et al*.* found that two genera of Proteobacteria, *Neisseria* and *Corynebacterium*, were enriched in samples from active EoE patients, while *Streptococcus* and *Atopobium* genera were more abundant in non-EoE controls. In our results, *Neisseria*, *Corynebacterium* and *Atopobium* all had a mean relative abundance below 1%, while a sizable number of samples had no presence of these genera (48%, 62% and 37%, respectively), thus limiting statistical comparisons. However, when these three genera were compared between samples from non-EoE controls and EoE patients at baseline our results coincided with those provided by Benitez et al*.* (being 0.59% *vs.* 1.01% for *Neisseria*, 0.09% *vs.* 0.19% for *Corynebacterium*, and 0.21% *vs.* 0.17% for *Atopobium*). For *Streptococcus* genus, we also observed a slightly higher abundance for controls compared to active EoE patients (42.9% *vs.* 40.6%) but this difference was far from being significant.

A second smaller study, published as poster communication, analyzed microbiota composition in biopsies from children with EoE^17^. Although some differences were observed between untreated EoE patients (n = 6) and controls (n = 18), no significant changes were found for specific taxa, with a trend towards decrease in the phylum Bacteroidetes in EoE. Conversely, our results showed greater abundance in Bacteroidetes in non-EoE controls and an increase in Proteobateria in EoE patients at baseline, which agreed better with the results described by Benitez et al.

The third study which analyzed microbiota in EoE patients was published by Harris *et al*^10^, who used an esophageal string test to obtain fluid samples from the esophageal secretions. The authors portray a decrease in Firmicutes and an increase in Proteobacteria in secretions from patients with active EoE, compared to controls and treated patients. We observed that Proteobacteria was also slightly higher in EoE baseline than in controls, while abundance of Firmicutes was similar in non-EoE controls and EoE baseline patients, but relatively increased in samples from EoE patients after PPI and FED therapies, and decreased in patients receiving STC. The only genus identified by Harris et al*.* as having a significantly greater abundance in samples from active EoE patients compared to controls was *Haemophilus*. Although *Haemophilus* was only detected in one of our samples, a non-significant increment was seen at baseline in the closely related *Actinobacillus* (mean relative abundance of 2.8% in controls *vs.* 8.9% in EoE baseline), the most prominent oral and esophageal species of which (*A. actinomycetemcomitans*) was reclassified to genus *Haemophilus* in 1985^18^. Later, *A. actinomycetemcomitans* and three *Haemophilus* genera were reclassified in the same genus, *Aggregatibacter*^19^, suggesting that both this work and the study by Harris observed an increase in EoE patients for these bacteria, but which were annotated differently in the microbial analysis. Its relative abundance was low in most patients (between 0–3%) but samples from some individuals showed high percentages over 15%, including one non-EoE control and eight baseline EoE samples. Other findings in this study agree more closely with Harris’s study, which also demonstrated no clear differences in bacterial alpha-diversity between controls and EoE patients, and cited differences in therapy undertaken as a source of variation in the composition of microbiomes of EoE patients recruited at several hospitals.

The microbiota composition of EoE has been also studied by using culture techniques instead of NGS in a series of 10 patients^20^. Evaluating only cultivable species and the low number of samples included in this research limits the conclusion of this study, which found that EoE patients showed more cultivable species in their esophagus than healthy volunteers and patients with gastroesophageal reflux disease (GERD).

It is believed that esophageal microbiota is quite similar to that in the oral cavity^7,21^, with its high prevalence of Streptococci and presence of genera such as *Prevotella*, *Veillonella*, *Gemella*, *Fusobacterium* and *Rothia* suggesting an oral origin for the esophageal microbiota. In addition, it might be influenced by migration of oral bacteria via swallowed or salivary secretions. Therefore, it makes sense to analyze the salivary microbiota in order to predict esophageal alterations in EoE^12^. The relative abundance of salivary *Haemophilus* in a series of 26 children with EoE correlated positively with validated EoE endoscopic and histological activity scores, suggesting the potential use of salivary microbiome as a non-invasive marker to monitor pediatric EoE.

The reduction of genus *Porphyromonas* in samples from active and treated EoE patients compared to control subjects was one of the significant differences found for microbial abundance in our study. One of its main species, *Porphyromonas gingivalis* is known by causing aggressive periodontitis^22^. It has been also involved recently with the pathogenesis of esophageal squamous cell cancer, its abundance correlating with cancer severity and poor prognosis^23^. Much less in know about *Parvimonas*, that was decreased in baseline EoE samples and its abundance recovered partially after EoE treatment. Its weight in terms of relative abundance was low as represented ~ 0.5% of non-EoE controls’ microbiota. Its only species, *Parvimonas micra*, is an oral anaerobic coccus that has been involved also in periodontitis in smokers^24^. Curiously, the third genus identified as less represented in EoE patients than in controls, *Filifactor*, is known to be involved in periodontitis as well^25^. Actually, it was described that one of its species, *Filifactor alocis*, forms a co-occurrence group with other potential pathogens (including two species of *Porphyromonas*) that was significantly enriched in periodontitis samples^26^. This observed relationship between a decrease in three genera associated with periodontitis and EoE may lead to further investigations of the role of these genera in healthy subjects or in other diseases of the upper gastrointestinal tract.

One of the main findings of our study was that different treatments for EoE induced different changes in esophageal microbiota according to beta-diversity analyses. The NMDS plot and clustering showed that STC differentiated microbial composition with respect to PPI and FED, which showed greater similarities among them. Grouping via DMM was based on observed differences in the distribution of “driver” taxa across the study in contrasting higher or lower abundances, before evaluating the ability of these newly defined cohorts to partition samples based on microbial composition. The two groups identified do not fully partition disease or treatment conditions, suggesting they may represent more basic differences in microbial community composition. Instead, this partitioning appears to represent modes of relatively low or high alpha-diversity (as shown by the corresponding gradient in Shannon diversity), in which case the co-localization of control and post-STC groups on the higher end of the alpha-diversity gradient is of interest.

Apart from compositional changes in bacterial taxa, our study provides some insight into enzymes and functions which might be altered in EoE, as well as being affected by the therapy applied. However, as the predictive method used has not been assessed previously in esophageal microbial samples, the results should be considered as putative differences in potential functions. Patients treated with STC are predicted to have higher microbial activity over that observed before treatment and in patients with FED/PPI therapies for oxidation/reduction of sulphur groups. Synthesis of peptidoglycan, a component of the bacterial cell wall involved in β-lactam resistance, was predicted to be more abundant in the microbiota of patients following FED than in those that underwent STC treatment. Microbiota of EoE patients were also predicted to have an increased abundance of arginine and ornithine synthesis pathways, which persisted for ornithine synthesis following STC therapy. Contrasting the impact of these changes, which is unclear in the literature, a presumed predicted reduction in microbial degradation of 4-aminobutanoate identified in patients with active EoE or following FED/PPI therapies could induce two direct effects in the esophagus: first, an increase in levels of gamma-aminobutyric acid (GABA) which is known to exert a role in esophageal motor function^27^; and second, a reduction in butanoate substrate which has anti-inflammatories properties^28^.

A limitation with our study is its relatively small sample size, although similar to cohorts recruited in previous studies about microbiota in EoE, which restricted outcomes and comparisons between treatment sub-groups. Given the high degree of variability of the microbiome observed in other locations (associated with factors such as health, environment, diet, and lifestyle), the enrolment of larger cohorts of EoE patients is required in future studies to accomplish a deeper analysis of oesophageal microbiota. Due to the limitation of low numbers of samples, we have included unadjusted *p*-values to inform about genera, enzymatic functions and metabolic pathways which were seen to change but did not reach statistical significance and therefore could be considered in future studies with larger sample sizes, or more exhaustive metabolic characterization. Moreover, as with most microbiota studies, our findings could be only interpreted as associations between microbial changes and EoE, without knowing whether they are causal, an effect of the disease, or due to intrapersonal variation or technical noise. Finally, other esophageal conditions were not considered. Consequently, we suggest that further studies including bigger cohorts of EoE patients and other esophageal diseases (GERD, Barret’s esophagus and esophageal carcinoma) should be performed in the future.

In conclusion, our findings support the idea that microbiota changes in EoE are modest but exist, and illustrate that reversion of those alterations is dependent on treatment, with STC leading to a microbiota composition more alike to non-EoE controls, and less similar to that of PPI and FED. We have also identified three genera previously unassociated with EoE, *Filifactor*, *Parvimonas* and *Porphyromonas*, whose abundance was decreased in baseline EoE patients. In addition, analysis of predicted function indicated that microbiota changes in EoE could lead to reduced abundance of sulphur-cytochrome oxidoreductases and disturbances in the metabolism of ornithine/arginine, peptidoglycan and amine-butyrate compounds.

## Methods

### Study participants and sampling

Patients with active EoE were prospectively recruited between 2017 and 2018. Diagnosis for EoE was defined by symptoms of esophageal dysfunction together with infiltration of esophageal epithelium by 15 or more eosinophil leukocytes per high-powered field (hpf). Eosinophilic infiltration in biopsy specimens from gastric and duodenal mucosa was excluded, as well as other causes of esophageal eosinophilia^1^. Patients were treated with anti-inflammatory drugs or diets according to clinical practice, and esophageal biopsy sampling was repeated after 6 to 12-weeks of therapy. Pairs of esophageal biopsy samples from 30 adult patients with active disease at baseline who achieved histological remission after 6 weeks of FED (10 patients), 8 weeks of double-dose PPI treatment (10 patients) or 12-weeks of STC (10 patients) were selected for this research.

In addition, control esophageal biopsy samples were obtained from 10 patients who underwent upper endoscopy mainly due to dyspepsia and exhibited a normal endoscopic appearance of the esophagus (hiatus hernia, incompetent cardias, and esophageal peptic lesions were excluded) and their esophageal biopsies were informed as normal.

All endoscopic exams were performed under propofol sedation by a single board-certified gastroenterologist (AJL) with a flexible 9.2-mm-caliber Olympus EXERA GIF-Q165 Video Gastroscope (Olympus Europe, Hamburg, Germany) and three biopsies were taken from both upper and lower esophageal thirds with the aid of a standard needle biopsy forceps (Endo Jaw FB-220U, Olympus Medical Systems, Tokyo, Japan). Apart from biopsies taken from the upper and lower esophageal thirds for histopathological assessment, three additional biopsies from the middle esophagus of each participant were collected and preserved in an RNA stabilization solution (RNAlater; Ambion, Austin, TX, USA) at –40 °C until processing.

Histological analyses were performed by an experienced pathologist (JMO). On each haemotoxylin and eosin-stained esophageal biopsy specimen the peak number of eosinophils was counted with the aid of Nikon Eclipse 50i (Nikon, Tokyo, Japan) light microscopy. All levels were surveyed and the eosinophils in the most densely infiltrated hpf (0.238 mm^2^) were reported as eosinophils/mm^2^.

The study was conducted in accordance with the principles of the Declaration of Helsinki and approved by the institutional review board of La Mancha Centro General Hospital. Informed consent was obtained from all patients at recruitment and prior to all endoscopic exams. For patients under 18, informed consent was obtained from a parent and/or legal guardian.

### DNA extraction

Biopsies preserved in RNAlater were sent on dry ice to the Institute of Pathology at Medical University of Graz (Graz, Austria) for DNA extraction and amplification for microbiota analyses. DNA extraction was performed using the XS buffer method^29^ with some modifications. XS buffer (2X) was freshly prepared as follows: (20 mL stock solution): 1 M Tris/HCl (pH 7.4) (4 mL); 7 M ammonium acetate (4.56 mL); 250 mM ethylene diamine tetraacetic acid (3.2 mL); 10% sodium dodecyl sulfate (w/v) (4 mL); potassium ethyl xanthogenate (0.4 g); and PCR-grade water (4.99 mL). For completely dissolving the xanthogenate, the buffer was incubated at 65ºC for 15 min.

After incubation at 65ºC for 30 min, biopsies were transferred to a Lysing Matrix E 2 mL tube (MP Biomedicals, Santa Ana, CA, USA) for sample homogenization via bead beating for a duration of 15 min. Then, sample was mixed with XS buffer (1:1 dilution) and incubated again at 65ºc for 2 h (mixing by hand every 30 min). Afterwards, the suspension was vortexed for 10 s, incubated on ice for 10 min and centrifuged (100 g, 5 min, 4ºC). The supernatant was transferred into a PhaseLock Gel tube (Eppendorf, Hamburg, Germany), and an equal volume of phenol: chloroform:isoamyl alcohol (25:24:1) was added. The suspension was mixed gently and centrifuged (2000 g, 5 min, 15ºC). The aqueous layer was transferred into a new tube. To precipitate DNA, the same volume of cold 100% isopropanol and 1/10 volume of 4 M ammonium acetate was added and gently mixed. After overnight incubation at –20ºC, the suspension was centrifuged (13,600 g, 30 min, 4ºC). The pellet was washed with 70% ethanol and centrifuged again. The pellet was dried completely and afterwards dissolved in 15 µL PCR-grade water.

### 16S rRNA gene PCR amplification and sequencing

Five microliters of total DNA (samples) or PCR-grade water (negative controls) were used in a 25 μL PCR reaction in triplicates using a FastStart High Fidelity PCR system (Roche, Mannheim, Germany). Each PCR reaction comprised of 1X Fast Start High Fidelity Buffer, 1.25 U High Fidelity Enzyme, 200 μM dNTPs, 0.4 μM primers and PCR-grade water (all products purchased from Roche). For amplification of the phylogenetically informative hypervariable region V4, the 16S rRNA primers 515F (GTGYCAGCMGCCGCGGTAA) and 806R (GGACTACNVGGGTWTCTAAT) were used with Illumina adapters for further indexing PCR reaction^30^.

Cycling conditions were 95ºC for 3 min followed by 30 cycles of 95ºC for 45 s, 55ºC for 45 s and 72ºC for 1 min, and a final extension step at 72ºC for 7 min^30^. Triplicates were pooled and checked on a 1% agarose gel before normalization of 20 μL PCR product on a SequalPrep Normalization Plate according to manufacturer’s instructions (LifeTechnologies, Darmstadt, Germany). Fifteen microliters of the normalized PCR product were used as template in a single 50 μL indexing PCR reaction for 8 cycles; the cycling conditions were as described above for the targeted PCR. Five microliters of PCR product from each sample were pooled to the final sequencing library and 30 μL were loaded to a 1% agarose gel for purification with the QIAquick gel extraction kit (Qiagen, Hilden, Germany). The purified library was quantified with QuantiFluor ONE dsDNA Dye on QuantusTM Fluorometer (Promega, Mannheim, Germany), loaded to an Agilent BioAnalyzer 2100 (Agilent, Waldbronn, Germany) for quality control and the 6 pM library was sequenced on a MiSeq desktop sequencer (Illumina, Eindhoven, Netherlands) containing 20% PhiX control DNA (Illumina) with v2 chemistry for 500 cycles. FASTQ raw reads were used for subsequent bioinformatic analysis.

### Bioinformatic analysis

All reads (300 bp paired-end, Illumina MiSeq) were quality-checked and trimmed at base position 19 (5′) and position 280 (3′), followed by terminal trimming of sequences with a quality (Q score) below Q = 24. Any sequences shorter than 150 bp were discarded before being imported into R using package DADA2^31^ to infer the unique amplicon sequence variants (ASV) representing different microbial populations. Samples were processed in parallel (maxEE of 3.4; reading 1 × 10^8^ bases from randomized reads) before pooling error profiles across the study and determining ASVs. Bimeras were removed in DADA2 before assigning taxonomic identities from the Silva database^32^ using the DECIPHER package^33^. Taxonomic identities were assigned below the rank of genus, i.e. to species-level where possible. Contaminant removal was carried out by comparison with negative controls and manual supervision: three *Streptococcus* ASVs were found in negative controls at very low abundances (40, 1, and 58 reads) but contributed the majority of all reads in the study (46%, 3% and 2% of all 622,379 reads): given their prevalence and established presence in the oesophagus^10^, these three ASVs were retained. Other ASVs found to be present in the negative controls (n = 44) were removed from the study. After contaminant removal, samples with fewer than 500 reads total were excluded (Supplementary Table 3 and Supplementary Fig. 6). Predicted metabolic content was generated using PICRUSt2^34^. Alpha-diversity, beta-diversity, and environmental gradients were explored in R (vegan functions diversity, estimate, monoMDS, ordisurf), with hierarchical clustering (Ward’s D2) of sample dissimilarity partitioned using R package DynamicTree Cut^35^ to produce sample clusters based on beta-diversity. Dirichlet multinomial modelling of population distributions was used to group samples based on the patterns of abundance underlying their community composition (R package DirichetMultinomial^36^), selecting the group number with the lowest Akaike information criterion (AIC).

### Statistical analysis

Alpha-diversity metrics were first rarefied to the lowest sample abundance (2013 reads) with 500 replicates (R package rtk)^37^. Rarefied alpha diversities were then evaluated using first the Shapiro–Wilk test for normality, followed by the Student’s t-test for normally distributed data, or the Mann–Whitney test when normality was absent. Paired t-test or Wilcoxon’s signed rank test were used when EoE baseline and post-treatment samples were compared. One way ANOVA was used to compare alpha-diversity among groups of patients split by the treatment received.

The total microbial composition of samples was assessed by first normalizing ASV features (geometric mean pairwise ratios, R package GMPR^38^), then converting to relative abundances, and calculating the Bray–Curtis dissimilarity. This dissimilarity was then used as input for non-metric dimensional scaling (NMDS, R package Vegan). To improve sensitivity to changes in abundance between features across samples of different size, a compositional data approach was adopted for both ASV and predicted EC features. Firstly, differences in amplicon library size were accommodated through normalization using GMPR^38^. Following this first step, ASV and EC abundances were agglomerated to genus and rank-3, respectively. Then, sparsity in the count abundance data was handled via a multiplicative replacement of zeroes (i.e. in each sample, zeroes were replaced with a pseudocount, followed by adjustment of all values in the sample by multiplication to maintain a constant sample sum^39^), before finally transforming the feature abundances via centered log-ratios (CLR). As taxonomic pathway and EC tables all comprised of different feature types, they were filtered separately based on their relative abundance and prevalence. This excluded taxa not present above 0.1% in 20% of all samples (66 genera), EC features not present above 10% abundance in 10% of samples (203 EC features), and pathways not present above 5% abundance in 5% of samples (281 pathways). Features were first screened using the Kruskal–Wallis test for differences in CLR abundance between groups (uncorrected *p*-value < 0.1), before testing of features that differed between groups using the Dunn test for unpaired two-way comparisons, and Wilcoxon’s signed rank test for paired two-way comparisons. Significance values in Dunn and Wilcoxon tests were adjusted via a Benjamini–Hochberg false discovery rate of 0.1 for ASV and EC abundances and of 0.25 for metabolic pathways.

## Supplementary Information

Supplementary Files

## Data Availability

Raw sequence data (including metadata) has been uploaded to the European Nucleotide Archive, and is available at https://www.ebi.ac.uk/ena/link, under the accession PRJEB39880 .

## Abbreviations

EoE: Eosinophilic esophagitis
FED: Empiric food-elimination diets
PPI: Proton pump inhibitors
STC: Swallowed topical corticosteroids

## References

[1] Lucendo AJ (2017). Guidelines on eosinophilic esophagitis: evidence-based statements and recommendations for diagnosis and management in children and adults. United Eur. Gastroenterol. J..

[2] Navarro P (2019). Systematic review with meta-analysis: the growing incidence and prevalence of eosinophilic oesophagitis in children and adults in population-based studies. Aliment. Pharmacol. Ther..

[3] Jensen ET, Dellon ES (2018). Environmental factors and eosinophilic esophagitis. J. Allergy Clin. Immunol..

[4] Jensen ET (2018). Early-life environmental exposures interact with genetic susceptibility variants in pediatric patients with eosinophilic esophagitis. J. Allergy Clin. Immunol..

[5] Pei Z (2004). Bacterial biota in the human distal esophagus. Proc. Natl. Acad. Sci. U.S.A..

[6] Yang L (2009). Inflammation and intestinal metaplasia of the distal esophagus are associated with alterations in the microbiome. Gastroenterology.

[7] Di Pilato V (2016). The esophageal microbiota in health and disease. Ann. N. Y. Acad. Sci..

[8] Corning B, Copland AP, Frye JW (2018). The esophageal microbiome in health and disease. Curr. Gastroenterol. Rep..

[9] Benitez AJ (2015). Inflammation-associated microbiota in pediatric eosinophilic esophagitis. Microbiome.

[10] Harris JK (2015). Esophageal microbiome in eosinophilic esophagitis. PLoS ONE.

[11] Dellon ES (2016). The esophageal microbiome in eosinophilic esophagitis. Gastroenterology.

[12] Hiremath G (2019). The salivary microbiome is altered in children with eosinophilic esophagitis and correlates with disease activity. Clin. Transl. Gastroenterol..

[13] Kashyap, P. C. *et al.* A decreased abundance of clostridia characterizes the gut microbiota in eosinophilic esophagitis. *Physiol Rep* **7**, (2019). 10.14814/phy2.14261 PMC6813259 31650712

[14] Proctor L (2019). A review of 10 years of human microbiome research activities at the US National Institutes of Health, Fiscal Years 2007–2016. Microbiome.

[15] Carding S, Verbeke K, Vipond DT, Corfe BM, Owen LJ (2015). Dysbiosis of the gut microbiota in disease. Microb. Ecol. Health Dis..

[16] Clooney AG (2016). Comparing apples and oranges?: Next generation sequencing and its impact on microbiome analysis. PLoS ONE.

[17] Smith, E. *et al.* Su1105 eosinophilic esophagitis: analyzing the esophageal and colonic microbiome. *Gastroenterology* **148**, S-409 (2015).

[18] Potts TV, Zambon JJ, Genco RJ (1985). Reassignment of Actinobacillus actinomycetemcomitans to the Genus Haemophilus as Haemophilus actinomycetemcomitans comb. nov. Int. J. Syst. Evol. Microbiol..

[19] Nørskov-Lauritsen N, Mogens K (2006). Reclassification of Actinobacillus actinomycetemcomitans, Haemophilus aphrophilus, Haemophilus paraphrophilus and Haemophilus segnis as Aggregatibacter actinomycetemcomitans gen. nov., comb. nov., Aggregatibacter aphrophilus comb. nov. and Aggregatibacter segnis comb. nov., and emended description of Aggregatibacter aphrophilus to include V factor-dependent and V factor-independent isolates. Int. J. Syst. Evol. Microbiol..

[20] NorderGrusell E, Dahlén G, Ruth M, Bergquist H, Bove M (2018). The cultivable bacterial flora of the esophagus in subjects with esophagitis. Scand. J. Gastroenterol..

[21] Wade WG (2013). The oral microbiome in health and disease. Pharmacol. Res..

[22] Nibali L (2015). Aggressive periodontitis: microbes and host response, who to blame?. Virulence.

[23] Gao S (2016). Presence of Porphyromonas gingivalis in esophagus and its association with the clinicopathological characteristics and survival in patients with esophageal cancer. Infect. Agents Cancer.

[24] Kumar PS (2012). Smoking and the subgingival ecosystem: a pathogen-enriched community. Fut. Microbiol..

[25] Moffatt CE (2011). Filifactor alocis interactions with gingival epithelial cells. Mol. Oral Microbiol..

[26] Chen H (2015). A Filifactor alocis-centered co-occurrence group associates with periodontitis across different oral habitats. Sci. Rep..

[27] Zhang Q, Lehmann A, Rigda R, Dent J, Holloway RH (2002). Control of transient lower oesophageal sphincter relaxations and reflux by the GABA(B) agonist baclofen in patients with gastro-oesophageal reflux disease. Gut.

[28] Gill PA, van Zelm MC, Muir JG, Gibson PR (2018). Review article: short chain fatty acids as potential therapeutic agents in human gastrointestinal and inflammatory disorders. Aliment. Pharmacol. Ther..

[29] Tillett D, Neilan BA (2000). Xanthogenate nucleic acid isolation from cultured and environmental cyanobacteria. J. Phycol..

[30] Kump P (2018). The taxonomic composition of the donor intestinal microbiota is a major factor influencing the efficacy of faecal microbiota transplantation in therapy refractory ulcerative colitis. Aliment Pharmacol. Ther..

[31] Callahan BJ (2016). DADA2: High-resolution sample inference from Illumina amplicon data. Nat. Meth..

[32] Quast C (2013). The SILVA ribosomal RNA gene database project: improved data processing and web-based tools. Nucleic Acids Res..

[33] Wright, E. S. Using DECIPHER v2.0 to Analyze big biological sequence data in R. *RJ* **8**, 352–359 (2016).

[34] Douglas, G. M. *et al.* PICRUSt2: An improved and customizable approach for metagenome inference. *bioRxiv* 672295 (2020). doi:10.1101/672295.

[35] Langfelder P, Zhang B, Horvath S (2008). Defining clusters from a hierarchical cluster tree: the dynamic tree cut package for R. Bioinformatics.

[36] Morgan, M. *DirichletMultinomial: Dirichlet-Multinomial Mixture Model Machine Learning for Microbiome Data*. (Bioconductor version: Release (3.11), 2020). doi:10.18129/B9.bioc.DirichletMultinomial.

[37] Saary P, Forslund K, Bork P, Hildebrand F (2017). RTK: efficient rarefaction analysis of large datasets. Bioinformatics.

[38] Chen L (2018). GMPR: A robust normalization method for zero-inflated count data with application to microbiome sequencing data. PeerJ.

[39] Martín-Fernández JA, Barceló-Vidal C, Pawlowsky-Glahn V (2003). Dealing with zeros and missing values in compositional data sets using nonparametric imputation. Math. Geol..

