# Supplementary material for: Esophageal microbiome in active eosinophilic esophagitis and changes induced by different therapies
Source: Sci Rep. 2021 Mar 29;11:7113. doi: 10.1038/s41598-021-86464-z (PMC8007638; doi:10.1038/s41598-021-86464-z)
Supplement: Supplementary file 1 — Supplementary Files [file 41598_2021_86464_MOESM1_ESM.docx]

**ESOPHAGEAL MICROBIOME IN ACTIVE EOSINOPHILIC ESOPHAGITIS AND CHANGES INDUCED BY DIFFERENT THERAPIES**

EJ Laserna-Mendieta^1,2,3,ǂ^, JA FitzGerald^4,5,ǂ^, L Arias-Gonzalez^1,2^, JM Ollala^6^, D Bernardo^7,8^, MJ Claesson^4,5^, AJ Lucendo^1,2,8,*^

1. Department of Gastroenterology. Hospital General de Tomelloso. Tomelloso, Spain
2. Instituto de Investigación Sanitaria de La Princesa. Madrid, Spain
3. Clinical Laboratory. Hospital Universitario de La Princesa. Madrid, Spain
4. School of Microbiology. University College Cork. Cork, Ireland
5. APC Microbiome Ireland. Cork, Ireland
6. Department of Pathology. Hospital General La Mancha Centro. Alcázar de San Juan, Spain
7. Mucosal Immunology Laboratory. Instituto de Biología y Genética Molecular (IBGM). Universidad de Valladolid. Valladolid, Spain
8. Centro de Investigación Biomédica en Red Enfermedades Hepáticas y Digestivas. Madrid, Spain

**ǂ EJ Laserna-Mendieta and Jamie Fitzgerald contributed equally to the first authorship.**

***Correspondence**

Emilio J Laserna Mendieta, PhD (ejlaserna@sescam.jccm.es)

Alfredo J Lucendo, MD, PhD, FEBGH (ajlucendo@hotmail.com)

Department of Gastroenterology, Hospital General de Tomelloso

Vereda de Socuéllamos, s/n, 13700 Tomelloso, Ciudad Real, Spain

Tel: + 34 926 525 927

Fax: + 34 926 525 870

**Supplementary Figure 1**. Comparison of oesophagus microbiota alpha diversity across treatment groups. Tukey plots showing alpha diversity for Chao1 (A and D), Shannon’s H (B and E) and inverse Simpson’s (C and F) indices. Comparison of paired pre- and post-treatment samples (n=26) for each of the indices analyzed (A-C). Comparison of baseline EoE samples divided by treatment for each of the indices analyzed (D-F). EoE eosinophilic oesophagitis; EoE post-Tx: EoE patients after treatment; FED: food elimination diet; PPI: proton pump inhibitors; STC: swallowed topical corticosteroids.

**Supplementary Figure 2.** Comparison of Faith’s phylogenetic diversity across treatment groups. Tukey plots showing Faith’s phylogenetic diversity. Comparison of controls and paired pre- and post-treatment samples (A). Comparison of samples divided by treatment received (B). EoE eosinophilic oesophagitis; EoE post-Tx: EoE patients after treatment; Neg control: negative controls for 16S amplification; FED: food elimination diet; PPI: proton pump inhibitors; STC: swallowed topical corticosteroids; *p < 0.05; **p < 0.01.

Supplementary Figure 3. Number of samples for each condition in the two Dirichlet multinomial mixtures (DMM) groups generated on the beta-diversity analysis using non-metric dimensional scaling (NMDS) (A); alpha-diversity measured by several indices (observed species, Shannon’s H and inverse Simpson’s) in each condition and divided according to DMM group, showing significant differences between the two groups (Kruskal-Wallis test, p < 0.0001) (B).


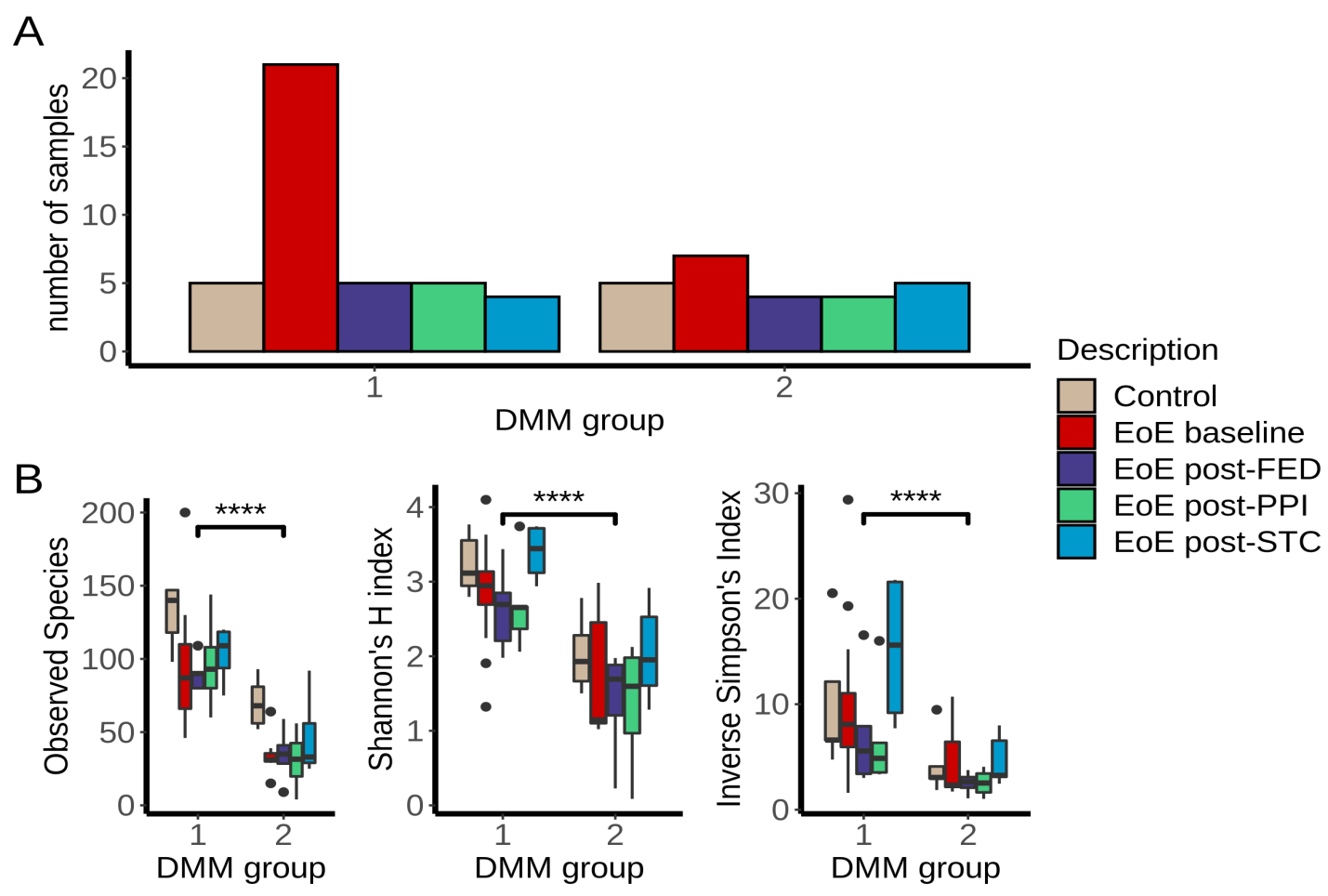


Supplementary Figure 4. Comparison of microbiome beta-diversity across conditions based on ASV features using principal co-ordinatates analysis (PCoA). Ellipses representing centroid position for each group, showing the distribution of samples and groups along the primary and secondary axes (pcoa1, pcoa3) (A); Dirichlet multinomial mixtures (DMM) groups defined through differences in patterns of abundance loosely separated along the primary X axis and gradient in alpha-diversity (B).


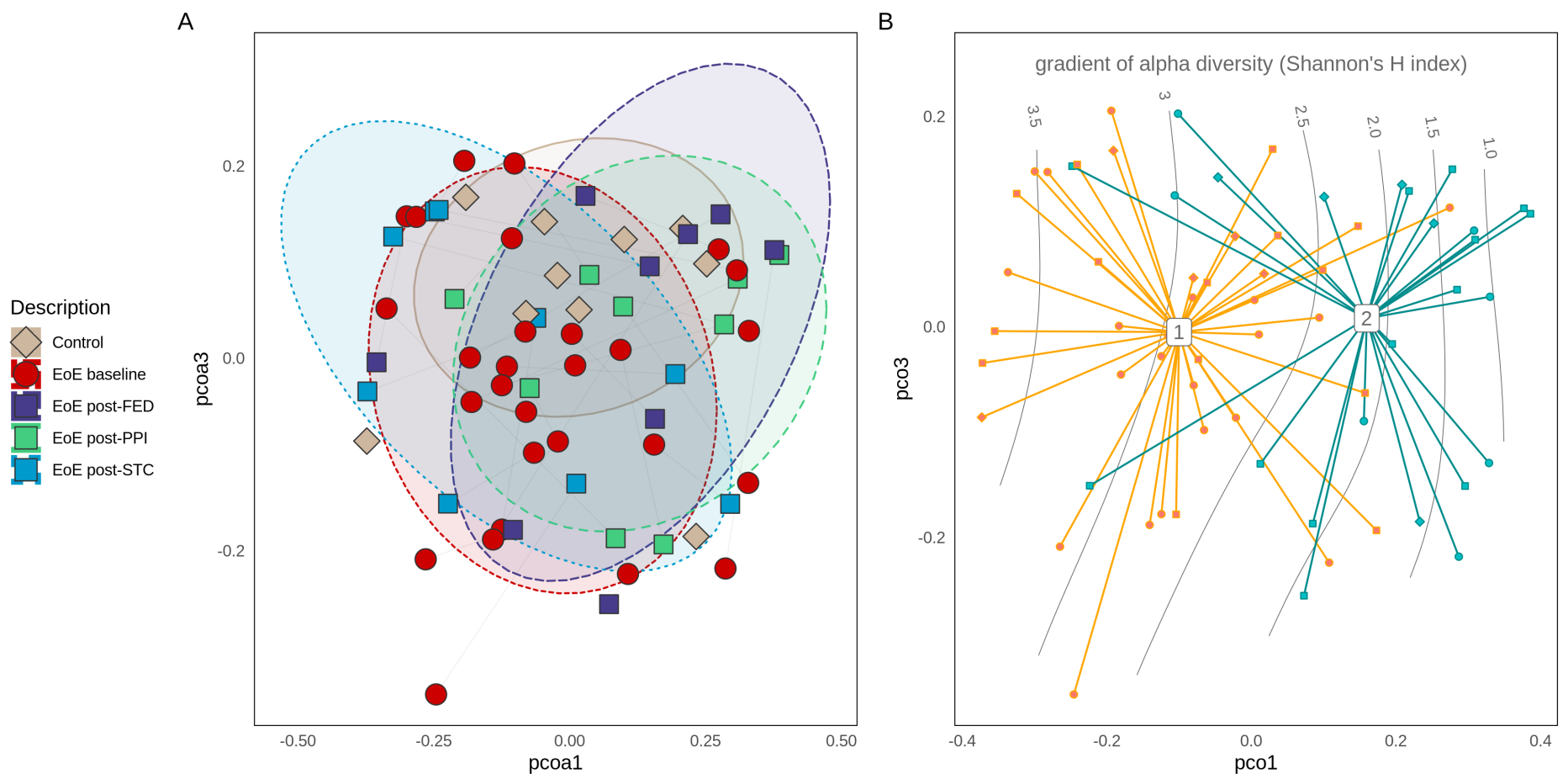


Supplementary Figure 5. Plots of predicted abundances transformed via CLR (centre log ratio) for EC (Enzymatic Commission Number) reactions and metabolic pathways. Only reactions (EC) and pathways (PWY) with at least one significant difference between groups according to adjusted p<0.1 and p<0.25 (Benjamini-Hochberg correction), respectively, are shown.


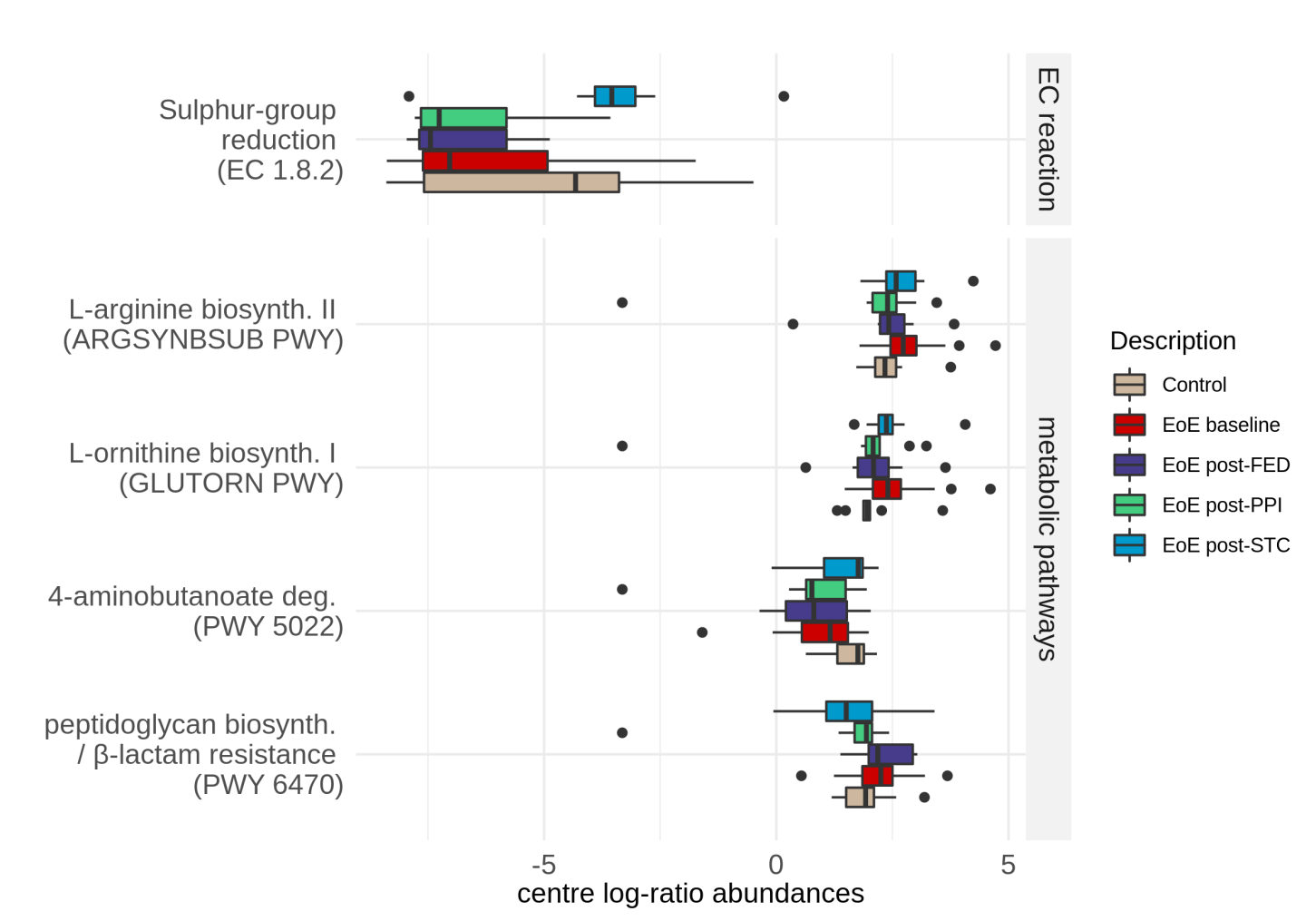


Supplementary Figure 6. Read count distribution after the final decontamination step. Samples with an asterisk were not included in the final analysis (having less than 500 reads).

Supplementary Table 1. Metadata of the patients and controls included in the study. EoE: eosinophilic oesophagitis; eos: eosinophils; Tx: treatment; NoR: final number of reads for microbiota analysis; FED: food-elimination diet; PPI: proton pump inhibitors; STC: swallowed topical corticosteroids; ND: not determined.

| Patient | EoE/control | Gender | Age | Tx (EoE)/ Cause for endoscopy (controls) | Specific Tx | Peak of eos  (pre-Tx) | Peak of eos  (post-Tx) | NoR (pre-Tx sample) | NoR (post-Tx sample) |
| --- | --- | --- | --- | --- | --- | --- | --- | --- | --- |
| 1 | EoE | Male | 21 | PPI | Lansoprazole | 85 | 3 | 30970 | 3482 |
| 2 | EoE | Male | 19 | STC | Budesonide | 20 | 0 | 1796 | 973 |
| 3 | EoE | Male | 45 | PPIs | Omeprazole | 30 | 6 | 8576 | 0 |
| 4 | EoE | Male | 16 | FED | Two-FED | 90 | 5 | 12501 | 8045 |
| 5 | EoE | Female | 32 | PPI | Omeprazole | 80 | 10 | 27401 | 1232 |
| 6 | EoE | Male | 36 | PPI | Omeprazole | 57 | 12 | 14199 | 14456 |
| 7 | EoE | Male | 31 | STC | Budesonide | 60 | 0 | 7783 | 6400 |
| 8 | EoE | Male | 44 | STC | Budesonide | 72 | 0 | 29483 | 26547 |
| 9 | EoE | Male | 35 | STC | Budesonide | 120 | 0 | 10934 | 3689 |
| 10 | EoE | Male | 42 | STC | Budesonide | 68 | 0 | 2209 | 352 |
| 11 | EoE | Male | 29 | STC | Budesonide | 95 | 0 | 12632 | 11580 |
| 12 | EoE | Male | 30 | STC | Budesonide | 20 | 0 | 3338 | 702 |
| 13 | EoE | Male | 18 | STC | Budesonide | 47 | 0 | 10262 | 8785 |
| 14 | EoE | Male | 16 | STC | Fluticasone | 135 | 0 | 9491 | 16744 |
| 15 | EoE | Male | 22 | STC | Budesonide | 65 | 0 | 5303 | 13994 |
| 16 | Control | Male | 50 | Dyspepsia | - | ND | | 15487 | |
| 17 | EoE | Male | 28 | PPI | Omeprazole | 50 | 3 | 920 | 11477 |
| 18 | EoE | Female | 23 | PPI | Omeprazole | 40 | 3 | 0 | 6515 |
| 19 | EoE | Male | 35 | PPI | Omeprazole | 54 | 0 | 3234 | 3662 |
| 20 | EoE | Male | 42 | PPI | Omeprazole | 25 | 0 | 12763 | 11479 |
| 21 | EoE | Male | 36 | FED | Four-FED | 100 | 3 | 9622 | 24516 |
| 22 | EoE | Male | 30 | PPI | Omeprazole | 90 | 0 | 9021 | 5027 |
| 23 | EoE | Male | 16 | FED | Six-FED | 40 | 1 | 225 | 142 |
| 24 | EoE | Male | 34 | FED | Two-FED | 110 | 15 | 11993 | 5043 |
| 25 | EoE | Male | 16 | FED | Two-FED | 100 | 3 | 31077 | 20236 |
| 26 | EoE | Male | 38 | FED | Two-FED | 157 | 5 | 11614 | 8572 |
| 27 | EoE | Male | 20 | FED | Four-FED | 160 | 0 | 6815 | 5022 |
| 28 | EoE | Female | 29 | FED | Four-FED | 80 | 0 | 13639 | 11298 |
| 29 | EoE | Male | 28 | FED | Two-FED | 185 | 15 | 10697 | 18668 |
| 30 | EoE | Male | 35 | FED | Four-FED | 70 | 0 | 6911 | 5490 |
| 31 | Control | Female | 39 | Dyspepsia | - | ND | | 7150 | |
| 32 | Control | Female | 27 | Dyspepsia | - | ND | | 17863 | |
| 33 | Control | Female | 29 | Dyspepsia | - | ND | | 18750 | |
| 34 | Control | Male | 21 | Dysphagia | - | 0 | | 22145 | |
| 35 | Control | Male | 35 | Dyspepsia | - | ND | | 18296 | |
| 36 | EoE | Male | 16 | PPI | Omeprazole | 60 | 0 | 7027 | 37480 |
| 37 | Control | Female | 53 | Iron deficiency | - | ND | | 24170 | |
| 38 | Control | Female | 16 | Dyspepsia | - | ND | | 2884 | |
| 39 | Control | Male | 41 | Iron deficiency | - | ND | | 17165 | |
| 40 | Control | Male | 48 | Dysphagia | - | 0 | | 11519 | |

Supplementary Table 2. Frequency table of Dirichlet Multinomial groupings over 10,000 bootstrapped hierarchical clustering steps. The table displayed the count of group members when polled at final consensus for the bootstrapping, as well as the boot mean stability (1 being fully stable, 0 being wholly unstable). EoE: eosinophilic oesophagitis; eos: eosinophils; FED: food-elimination diet; PPI: proton pump inhibitors; STC: swallowed topical corticosteroids.

|  | **Cluster assignment** | |
| --- | --- | --- |
|  | **Arm A** | **Arm B** |
| Mean stability | 0.896 | 0.860 |
| Control | 7 | 3 |
| EoE Baseline | 17 | 11 |
| EoE post-FED | 8 | 1 |
| EoE post-PPI | 8 | 1 |
| EoE post-STC | 3 | 6 |
| **Total** | **43** | **22** |

Supplementary Table 3. Number of reads per sample after each step of removal of reads before data analysis. Last column displays the final number of ASV per sample. ASV: amplicon sequence variants; C: control sample; POST: sample after treatment; PRE: baseline sample before treatment. Samples with asterisk were not included in the analysis.

| **Sample** | **raw** | **trimmed** | **filtered** | **denoised** | **ASV assigment** | **ASV final** |
| --- | --- | --- | --- | --- | --- | --- |
| 01POST | 4446 | 4440 | 3792 | 3632 | 3482 | 56 |
| 01PRE | 37427 | 37398 | 33834 | 31430 | 30970 | 200 |
| 02POST | 1820 | 1819 | 1395 | 1223 | 973 | 33 |
| 02PRE | 2269 | 2261 | 1987 | 1850 | 1796 | 54 |
| 03POST* | Not sequenced | | | | | |
| 03PRE | 10089 | 10080 | 9100 | 8744 | 8576 | 124 |
| 04POST | 9355 | 9343 | 8336 | 8217 | 8045 | 59 |
| 04PRE | 14824 | 14810 | 13452 | 12681 | 12501 | 88 |
| 05POST | 2114 | 2112 | 1700 | 1588 | 1232 | 25 |
| 05PRE | 33752 | 33731 | 30744 | 27655 | 27401 | 122 |
| 06POST | 17426 | 17415 | 15853 | 14536 | 14456 | 93 |
| 06PRE | 17479 | 17464 | 15759 | 14344 | 14199 | 114 |
| 07POST | 7818 | 7809 | 6938 | 6805 | 6400 | 92 |
| 07PRE | 9506 | 9500 | 8595 | 7839 | 7783 | 80 |
| 08POST | 37029 | 37005 | 34119 | 26710 | 26547 | 75 |
| 08PRE | 35793 | 35772 | 32583 | 29595 | 29483 | 32 |
| 09POST | 4987 | 4981 | 4272 | 4102 | 3689 | 100 |
| 09PRE | 13339 | 13324 | 11882 | 11495 | 10934 | 110 |
| 10POST* | 1028 | 1028 | 783 | 758 | 352 | * |
| 10PRE | 3027 | 3024 | 2474 | 2387 | 2209 | 51 |
| 11POST | 13138 | 13121 | 11940 | 11728 | 11580 | 120 |
| 11PRE | 15246 | 15234 | 13888 | 12683 | 12632 | 29 |
| 12POST | 1106 | 1106 | 884 | 815 | 702 | 25 |
| 12PRE | 4186 | 4180 | 3616 | 3409 | 3338 | 52 |
| 13POST | 10091 | 10083 | 9211 | 8965 | 8785 | 56 |
| 13PRE | 12150 | 12139 | 10873 | 10334 | 10262 | 76 |
| 14POST | 20096 | 20083 | 18545 | 16811 | 16744 | 29 |
| 14PRE | 11192 | 11182 | 9903 | 9735 | 9491 | 64 |
| 15POST | 17224 | 17208 | 15751 | 14095 | 13994 | 118 |
| 15PRE | 6027 | 6022 | 5517 | 5365 | 5303 | 46 |
| 16C | 18344 | 18338 | 16715 | 15639 | 15487 | 98 |
| 17POST | 13644 | 13632 | 12155 | 11653 | 11477 | 144 |
| 17PRE | 1465 | 1463 | 1273 | 1153 | 920 | 39 |
| 18POST | 7578 | 7563 | 6686 | 6657 | 6515 | 4 |
| 18PRE* | 62 | 61 | 11 | 0 | 0 | * |
| 19POST | 4296 | 4294 | 3844 | 3750 | 3662 | 38 |
| 19PRE | 3707 | 3705 | 3358 | 3283 | 3234 | 68 |
| 20POST | 12862 | 12859 | 11731 | 11603 | 11479 | 80 |
| 20PRE | 14729 | 14715 | 13265 | 12872 | 12763 | 31 |
| 21POST | 30285 | 30263 | 27320 | 24740 | 24516 | 90 |
| 21PRE | 11354 | 11348 | 10261 | 9704 | 9622 | 73 |
| 22POST | 5976 | 5974 | 5463 | 5236 | 5027 | 60 |
| 22PRE | 10792 | 10786 | 9692 | 9469 | 9021 | 66 |
| 23POST* | 466 | 466 | 337 | 309 | 142 | * |
| 23PRE* | 345 | 345 | 306 | 275 | 225 | * |
| 24POST | 5602 | 5600 | 5195 | 5101 | 5043 | 35 |
| 24PRE | 13846 | 13842 | 12808 | 12127 | 11993 | 65 |
| 25POST | 23733 | 23712 | 21523 | 21391 | 20236 | 90 |
| 25PRE | 37878 | 37861 | 34829 | 31314 | 31077 | 130 |
| 26POST | 10196 | 10188 | 9168 | 8650 | 8572 | 35 |
| 26PRE | 14911 | 14901 | 13278 | 11739 | 11614 | 104 |
| 27POST | 6079 | 6075 | 5410 | 5244 | 5022 | 80 |
| 27PRE | 8559 | 8551 | 7714 | 6879 | 6815 | 103 |
| 28POST | 13387 | 13377 | 12100 | 11440 | 11298 | 109 |
| 28PRE | 16522 | 16513 | 14536 | 14441 | 13639 | 87 |
| 29POST | 21679 | 21657 | 19605 | 19075 | 18668 | 80 |
| 29PRE | 13223 | 13218 | 11977 | 10782 | 10697 | 94 |
| 30POST | 6197 | 6194 | 5600 | 5546 | 5490 | 9 |
| 30PRE | 7767 | 7759 | 7131 | 6939 | 6911 | 15 |
| 31C | 8571 | 8568 | 7664 | 7523 | 7150 | 56 |
| 32C | 20275 | 20269 | 18523 | 18036 | 17863 | 147 |
| 33C | 22796 | 22779 | 20847 | 20211 | 18750 | 140 |
| 34C | 26414 | 26398 | 24064 | 22332 | 22145 | 81 |
| 35C | 21092 | 21075 | 19064 | 18631 | 18296 | 118 |
| 36POST | 48559 | 48528 | 44379 | 37729 | 37480 | 108 |
| 36PRE | 36422 | 36318 | 33671 | 33532 | 7027 | 31 |
| 37C | 33060 | 33037 | 29785 | 24298 | 24170 | 93 |
| 38C | 4228 | 4224 | 3489 | 3320 | 2884 | 52 |
| 39C | 21345 | 21331 | 19303 | 17310 | 17165 | 147 |
| 40C | 13491 | 13483 | 12306 | 11554 | 11519 | 68 |
| Negative C1 | 18384 | 18319 | 16416 | 16311 | 0 | * |
| Negative C2 | 31476 | 31410 | 29368 | 29187 | 0 | * |
| Negative C3 | 17020 | 16902 | 15664 | 15301 | 0 | * |
